# Mebendazole stimulates CD14+ myeloid cells to enhance T-cell activation and tumour cell killing

**DOI:** 10.18632/oncotarget.25713

**Published:** 2018-07-20

**Authors:** Jenny Rubin, Sharmineh Mansoori, Kristin Blom, Malin Berglund, Lena Lenhammar, Claes Andersson, Angelica Loskog, Mårten Fryknäs, Peter Nygren, Rolf Larsson

**Affiliations:** ^1^ Department of Medical Sciences, Division of Cancer Pharmacology and Computational Medicine, Uppsala University, Uppsala, SE-75185, Sweden; ^2^ Department of Immunology, Genetics and Pathology, Uppsala University, Uppsala, SE-75185, Sweden

**Keywords:** mebendazole, immune activation, drug repositioning, cancer

## Abstract

Mebendazole (MBZ) was recently shown to induce a tumor suppressive M1 phenotype in THP-1 monocytes and macrophages. In the present study the immune effects of MBZ was further investigated using human peripheral blood mononuclear cells (PBMCs) co-cultured with tumour cells. The Biomap platform was used to screen for biomarkers induced from MBZ exposed co-cultures of T-cell receptor activated PBMCs, HT29 colon cancer cells and either human fibroblasts or human umbilical vein endothelial cells (HUVEC) cells. In these co-culture systems MBZ at 0.3-10 μM induced significant increases in TNFα and IFNγ indicating immune stimulation. PBMC cultures alone were subsequently tested for activation status and only in PBMCs activated by CD3/IL2 stimulation and MBZ, at a clinically achievable concentration, was able to increase PBMC clustering and release of pro-inflammatory IFNγ, TNFα, IL6 and IL1β cytokines. Moreover, when PBMC cultures were functionally tested for immune cell killing of lung cancer A549NucLightRed cells, MBZ significantly increased tumour cell apoptosis and reduced the number of surviving tumour cells. This effect was dependent on the presence of CD14 monocytes/macrophages in the co-culture. In summary, MBZ potentiated the immune stimulatory and anticancer effects of anti-CD3/IL2 activated PBMCs which could be relevant to explain the anticancer activity of MBZ observed in the clinic.

## INTRODUCTION

The monocyte-macrophage system display at least two distinct phenotypes of differentiation being pro-inflammatory (M1) or anti-inflammatory (M2) [1–3]. The M1 monocytes/macrophages have phagocytic and antigen-presenting activity, produce Th-1 activating cytokines, and induce cytotoxic effects [1–3]. M1 macrophages can also activate other cells of the immune system, such as natural killer (NK) and T lymphocytes. The M2 macrophages/monocytes, on the other hand, are thought to induce tumor-supporting, angiogenic and immunosuppressive effects and are the dominant myeloid phenotype infiltrating solid tumours [1–3]. New drugs that can repolarize tumor associated M2 macrophages to the tumor suppressive M1 phenotype would be of great importance, especially for tumours with high numbers of myeloid cells but few T-cells (so called “cold” tumours). This type of drugs could also be a useful complement as sensitisers to other immunotherapies such as checkpoint inhibitors.

Mebendazole (MBZ) is clinically used to treat various forms of helminthic diseases but has also been reported to demonstrate anticancer activity in models both *in vitro* and *in vivo* [4–14]. MBZ has also produced objective tumour responses in therapy-resistant cancer patients in the clinical setting [15, 16]. The anticancer properties of MBZ has long been attributed to its ability to target and inhibit tubulin polymerization [6, 7]. However, other directly tumour cell related mechanisms, including protein kinase inhibition [10], anti-angiogenesis [9, 12], pro-apoptotic activity [5, 11], and inhibition of the Hedgehog pathway [17] have been proposed.

Recently, we demonstrated that MBZ induce a pro-inflammatory tumour-suppressive M1 phenotype in THP-1 monocytes and macrophages. MBZ-induced IL1β release was found to be dependent on NLRP3 inflammasome activation and to involve toll-like receptor 8 (TLR8) stimulation [18].

In the present study we investigated further the immune modulating properties and anticancer properties of MBZ in PBMCs co-cultured with normal and/or tumour cells. We demonstrate that MBZ at clinically achievable concentration potentiated the anticancer activity of CD3/IL2 activated PBMCs and that this effect was attenuated by removal of CD14 positive cells.

## RESULTS AND DISCUSSION

**Figure 1:**
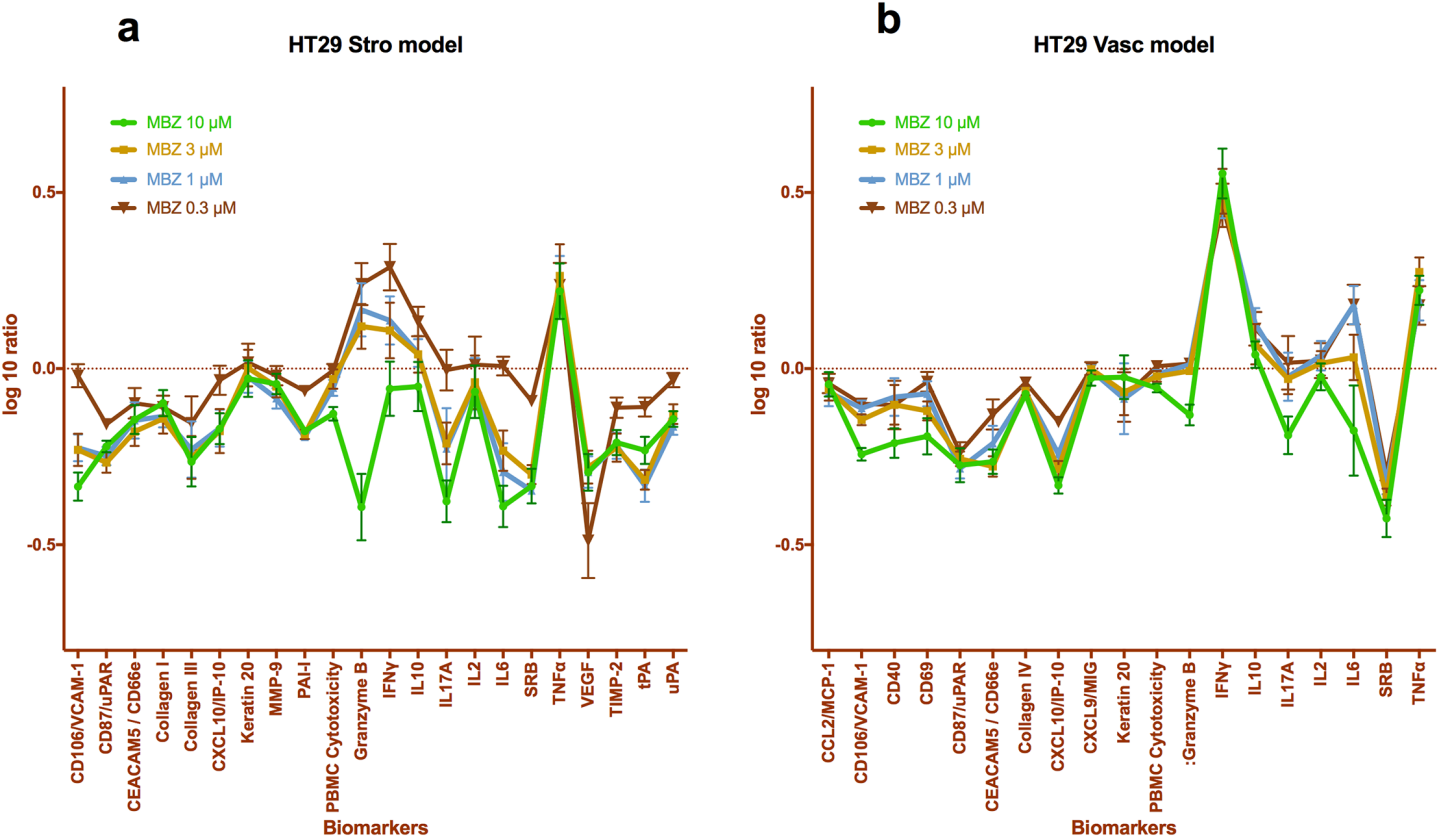
Biomap profiles of MBZ, tested at multiple concentrations in 2 BioMAP systems, HT29 Vasc **(a)** and HT29 Stro **(b)**. The biomarker readouts measured (see Methods) are indicated along the x-axis. The y-axis shows the log10 expression ratios of the readout level measurements relative to solvent (DMSO buffer) controls.

To further explore the immunomodulating properties of MBZ we took advantage of the Biomap platform (DiscoverX). In this assay system tumour cells (HT29) and SAg activated PBMCs are co-cultured with either primary human fibroblasts (Stro model) or HUVEC (Vasc model) cells. MBZ at concentrations between 0.3 and 10 μM significantly increased the levels of Granzyme B (Figure 1a), TNFα and IFNγ (Figure 1a, 1b) with a concomitant decrease in VEGF (Figure 1a) and SRB (Figure 1a, 1b). These results exceeded the 95% confidence interval of DMSO treated controls. The SRB decrease is probably due to inhibition of tumour cell growth since in MBZ treated co-cultures with fibroblasts and activated PBMC alone SRB was not decreased (Supplementary Figure 1). Also, levels of VCAM-1, Collagen III, IL6 and tPA (Figure 1a) and CD87/uPAR and CXCL10/IP-10 (Figure 1b) were significantly reduced from control for most MBZ concentrations tested. The Biomap platform has been shown to deliver robust profiling of drugs from different classes with respect to both toxicity and mechanism of action [19].

**Figure 2:**
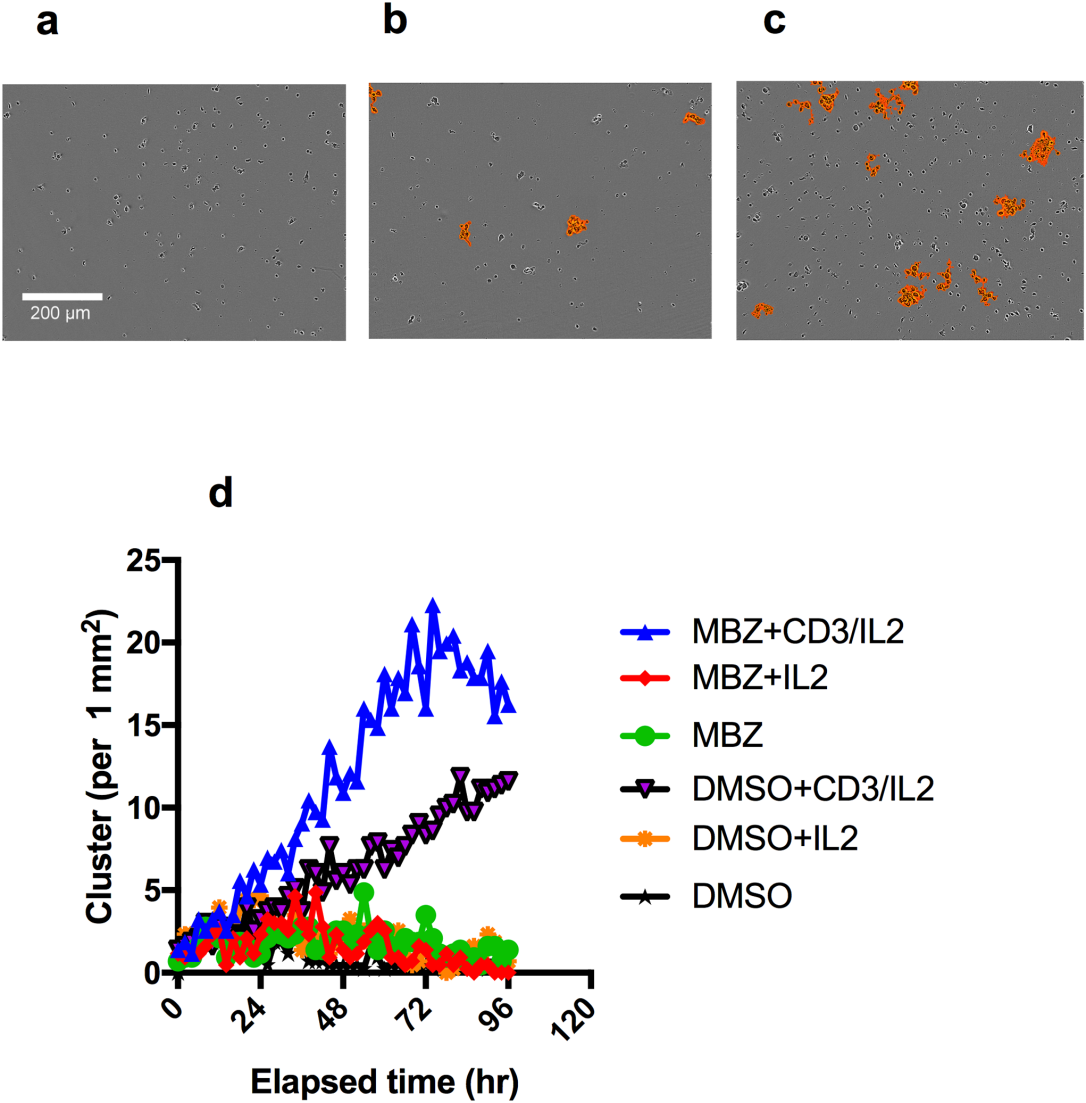
Clustering of PBMC cultures in response to anti-CD3/IL2 and MBZ Photomicrographs shows the effect of CD3/IL2 (0.5/2.5 μM) alone **(a)** together with 1 μM MBZ **(b)** in comparison with a DMSO exposed control **(c)** on clustering assessed in the Incucyte Zoom. An orange mask was added to visualise the the criteria used by the Incucyte to define the clusters. In **(d)** the kinetics of clustering over time is shown. The experiment was repeated at least 3 times with similar results.

To verify and complement these data we tested the activity of MBZ on clustering and proliferation of PBMCs activated through the CD3 molecule of the T-cell receptor complex. This test was performed at a clinically relevant concentration of 1 μM [20]. MBZ showed no increased clustering in unexposed PBMC (Figure 2a, 2b, 2d) but clearly increased clustering was noted in CD3/IL2 activated PBMCs (Figure 2c, 2d). The image based clustering assay is a simple assay to monitor immune cell activation. During an immune response, activated cells of the immune system, such as T-cells, undergo rapid expansion and many interactions also occur between activated immune cells (e.g., T cell interactions with antigen-presenting cells and interactions between T cells themselves). These dynamic changes in cell-cell interactions can easily be captured by the Incucyte clustering assay.

**Figure 3:**
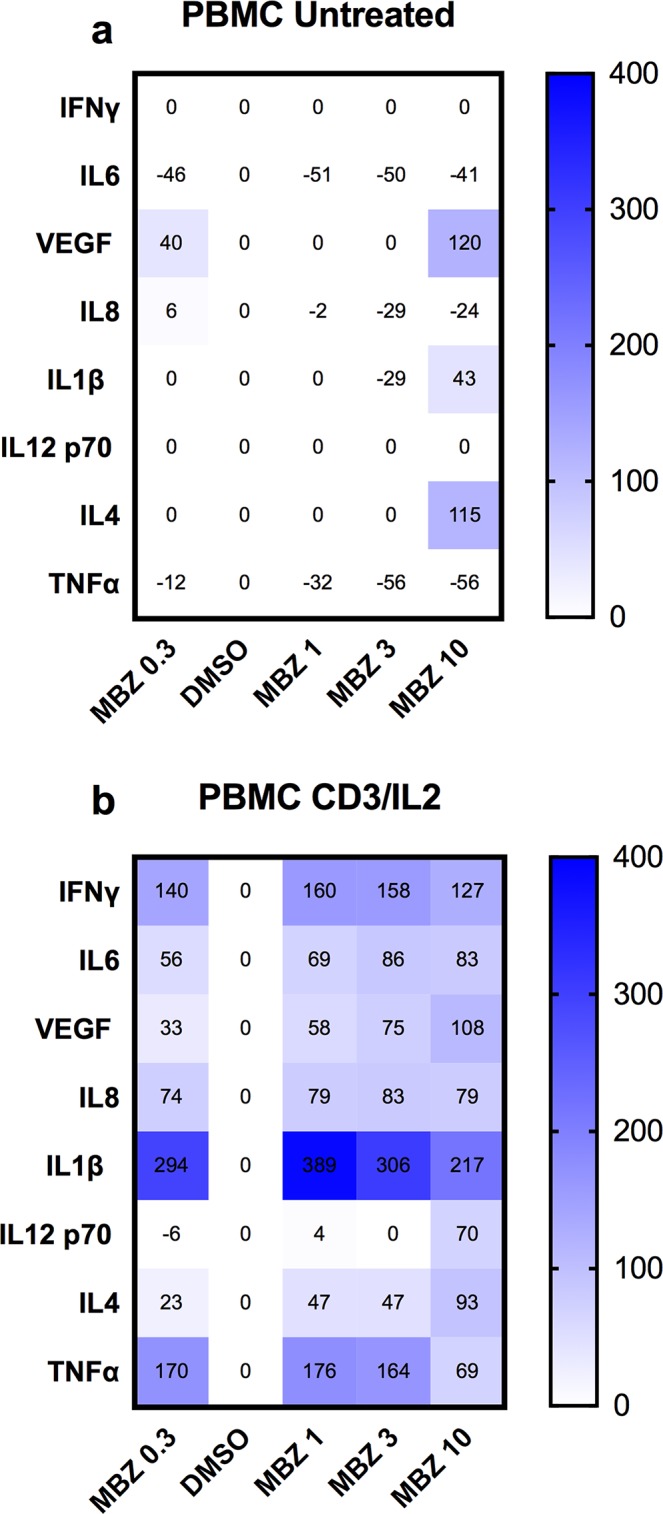
Cytokine release from PBMCs in response to 24 h exposure to the indicated concentrations of MBZ relative to DMSO control The effect of increasing concentrations of MBZ on unexposed PBMCs **(a)** or those exposed to anti-CD3/IL2 **(b)** is shown using a multiplex kit from R&D. Results are presented as % over DMSO (0.1%) control. A value of zero includes data where both DMSO and MBZ treatment did not reach the lowest concentration of the standard curves. The experiment was repeated three times with similar results.

Next we tested the ability of MBZ to induce cytokine release from PBMC cultures. Again, MBZ at 1-10 μM had little or no effect on cytokine release from non-activated PBMCs (Figure 3a) but stimulated the release of several pro-inflammatory cytokines including TNFα, IL1β, IFNγ, IL6 from PBMCs activated by IL2 and anti-CD3 stimulation (Figure 3b). This has clear resemblance with our previous study in which MBZ at low concentrations require priming with LPS to stimulate pro-inflammatory cytokine (primarily IL1β) release in the THP-1 model [18]. IL1β secretion is suggested to require two signals. The first is provided by toll-like-receptor-mediated NF-kappaB activation whereas the second can be mediated by danger signals such as stimulation of ATP receptors or other stimuli including elevated ROS production or perturbation of lysosome integrity. In our previous study it was demonstrated that high dose MBZ (>3 μM) activated both signals whereas low concentrations (1 μM) only could activate the second one [18].

**Figure 4:**
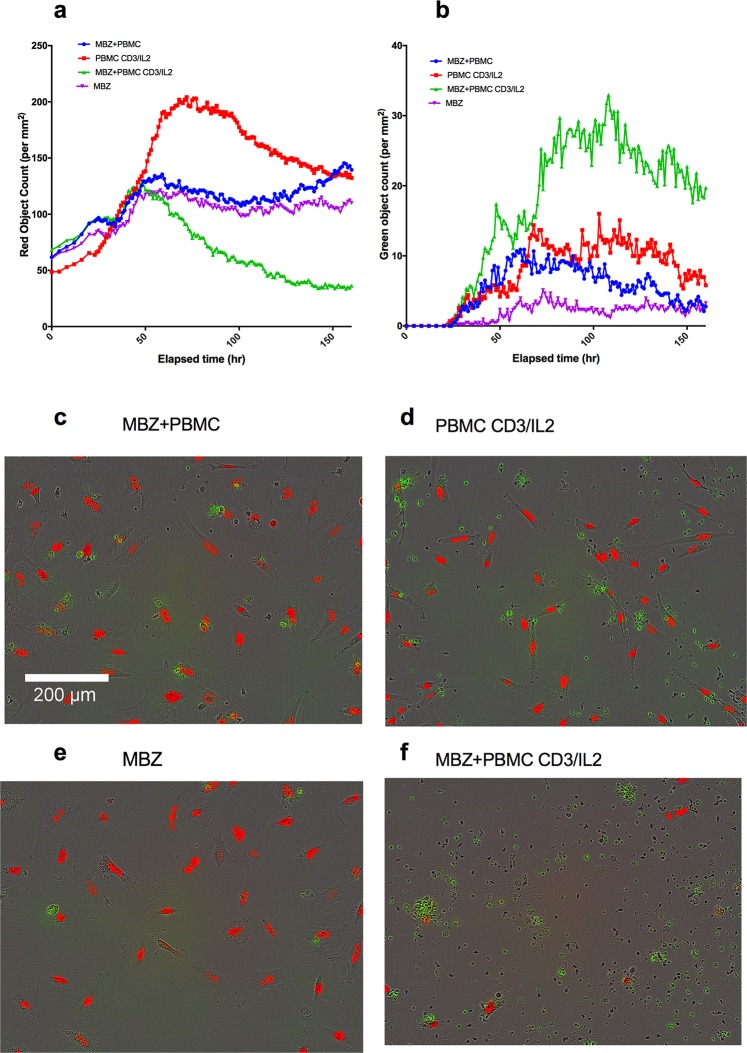
Co-culture of PBMCs and red fluorescence-labeled A549 lung cancer cells in medium containing caspase 3/7 probe (Cas3/7) The kinetics of changes in A549 cell survival (red object count) and apoptosis (green object count, cas3/7 positive cells) in response to anti-CD3/IL2 and MBZ is presented in panel **(a** and **b)**, respectively. Representative photomicrographs for the different incubations after 1 week are shown in **(c-f)**, as indicated. The difference in red object count measured as AUC between the MBZ+CD3/IL2 treatment group vs all other groups including MBZ without PBMC (see Table 1). This was also the case when comparing AUC of green object counts (caspase positive cells).

**Figure 5:**
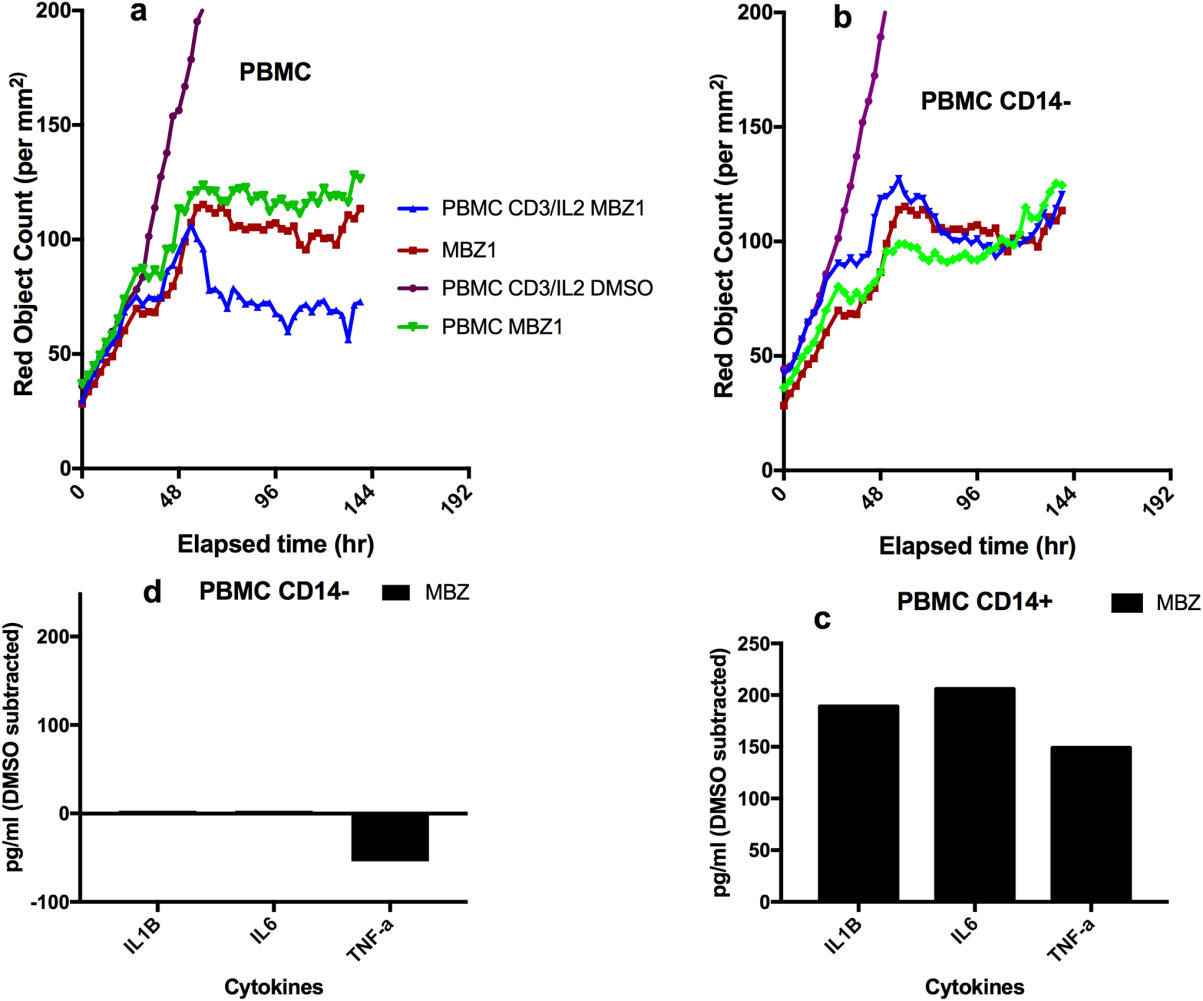
Effect of depletion of CD14+ cells on MBZ induced potentiation of anti-CD3/IL2 induced PBMC mediated inhibition of A549 tumor cell survival and cytokine release In upper panels the effect of intact non-depleted PBMC **(a)** and PBMCs depleted of CD14 cells **(b)** on A549 cell proliferation (red object count) is shown over time. Panel **(c** and **d)** shows the cytokine release for the corresponding PBMC cultures after 24 h of incubation. Statistical analysis is shown in Table 1.

Finally, we tested the ability of MBZ to impact on immune cell killing in co-cultures of PBMCs with tumour cells (A549, Figure 4a-4f). Again, in CD3/IL2 stimulated cells, MBZ (1 μM) potentiated both tumour cell reduction (Figure 4a, 4d, 4f) and tumour cell apoptosis (Figure 4b, 4d, 4f) whereas no apparent effect was observed in non-stimulated cultures (Figure 4a, 4b, 4c, 4e). Statistical analysis of the differences between groups are shown in Table 1 (Paired t-tests n=10). Very similar results were observed in response to MBZ at 10 μM (Supplementary Figure 2) Corresponding statistical analysis for MBZ 10 μM is shown in Supplementary Table 1. Moreover, the effect seemed to be dependent on the presence of monocytes and macrophages since removal of CD14 expressing cells diminished the anti-tumour activity of MBZ (Figure 5a, 5b, Table 1, n=3). This was also evident when measuring cytokine release. In CD14 depleted PBMC cultures MBZ induced a diminished release of IL1β, TNFα and IL6 compared to intact PBMC cultures (Figure 5c, 5d) Removal of CD56+ cells had no effect whereas a modest attenuation of MBZ+CD3/IL2 anti-tumor activity was observed after depleting CD8+ cells (Supplementary Figure 3). These results are in accordance with the hypothesis that MBZ acts via monocyte/macrophage activation and polarization towards the M1 phenotype [18].

**Table 1:** Statistical analysis of the differences in AUC between groups (MBZ 1 μM)

Object (AUC)	Comparison	Mean Difference	P-value	N	PVS^1^
**Red**	DMSO+PBMC CD3/IL2 vs. MBZ+PBMC CD3/IL2	28934	0.0003	10	^***^
**Red**	MBZ+PBMC vs. MBZ+PBMC CD3/IL2	5196	0.0028	10	^**^
**Red**	MBZ vs. MBZ+PBMC CD3/IL2	5565	0.0007	10	^***^
**Green**	DMSO+PBMC CD3/IL2 vs. MBZ+PBMC CD3/IL2	1967	0.0065	10	^**^
**Green**	MBZ+PBMC vs. MBZ+PBMC CD3/IL2	4472	0.0050	10	^**^
**Green**	MBZ vs. MBZ+PBMC CD3/IL2	4957	0.0022	10	^**^
**Red**	MBZ+PBMC CD3/IL2 vs. MBZ+PBMC (−CD14) CD3/IL2	93.67	0.0006	3	^***^

Data was analysed by two-tailed paired t test using Graphpad Prism. ^1^P value summary.

The exact mechanism for this potentiation is not clear but likely involves MBZ induced cytokine release (TNFα, TRAIL) by monocytes/macrophages [21] and/or indirect stimulation of immune cell interactions leading to enhanced T-cell or NK cell mediated anti-tumour responses [22]. However, a contribution from MBZ induced tubulin inhibition cannot be excluded. Indeed, several tubulin inhibitors have been reported to stimulate immune responses in dendritic cells [23]. For example, vinblastine, a prototypic drug of this class, induced the production of IL1β, IL6, and IL12, increased surface expression of CD40, CD80, CD86, and augmented T cell–stimulatory capacity of DCs [23, 24]. On the other hand, it was previously shown that MBZ, but not other tubulin inhibitors, could induce cytokine release in monocytic models [18, 25]. Neverheless, the exact role of tubulin interactions in mediating MBZ induced tumour suppression in the co-culture system remains to be elucidated and the mechanism may be multifactorial.

Taken together, our data indicates that MBZ could be an interesting drug candidate to modulate the myeloid cell linage in cancer patients aiming to support anti-tumour immunity via T-cell activation.

## MATERIALS AND METHODS

### *In vitro* models and cell culture

The human A549NucLightRed Cell line (Essen Bioscience cat # 4491) (Essen Bioscience, Ann Arbor, MI, USA) was cultured in Ham's F-12 Nutrient Mix, GlutaMAX Supplement, (Gibco cat # 31765-027) (ThermoFisher Scientific, Waltham, MA, USA), supplemented with 10% heat-inactivated fetal bovine serum (Sigma F9665) (Sigma-Aldrich, Munich, Germany), 100 g/mL streptomycin, 100 U/mL penicillin (Sigma P0781) and 0,5 g/mL Puromycin (Sigma P9620). The cell line was kept at 37°C in a humidified incubator supplied by 5% CO_2_. The cell line was splitted twice a week and kept for a maximum of 20 passages. Morphology and growth of cells were monitored on a weekly basis. Peripheral blood mononuclear cells (PBMC) from healthy donors were isolated by Ficoll Histopaque (Sigma) density gradient centrifugation. In some experiments CD14 expressing cells were removed from the PBMC preparation. CD14+ monocytes were removed by positive selection using CD14 MicroBeads (Miltenyi Biotec, Bergisch Gladbach, Germany) on an AutoMACS Pro Separator (Miltenyi Biotec). The purity of the eluted monocyte preparations was assessed by flow cytometry using a Navios Flow Cytometer (Beckman Coulter, Indianapolis, IN, USA).

### Materials

MBZ was purchased from Sigma. The compound was kept as 10 mM stock solutions in dimethyl sulphoxide (DMSO, Sigma) and further diluted with culture medium (Sigma or ATCC) as needed. Anti-human CD3 was purchased from Affymetrix (Santa Clara, CA, USA) and IL-2 was purchased from Peprotech (Rocky Hill, NJ, USA). All other reagents used were obtained from Sigma.

### Biomap analysis

To determine the activity of MBZ in a complex primary culture human cell systems, we tested the compound at 0.3, 1, 3 and 10 μM in the BioMAP Oncology and Immune Oncology PanelsSystems (DiscoverX, CA, USA). These systems consist of complex co-cultures of PBMCs pooled from healthy donors with early passage human primary fibroblasts or endothelial cells, with or without a tumor cell line and are stimulated to recapitulate and model tumor-immune-stromal (StroHT29), or tumour-immune-vascular (VascHT29) microenvironments. The StroHT29 consists of primary fibroblasts co-cultured with the adenocarcinoma cell line HT-29 and human PBMCs stimulated via the T-cell receptor by SAg (superantigen) for 48 h. The VascHT29 system is identical except for primary human umbilical vein endothelial cells (HUVEC) replacing the fibroblasts.

Compounds were prepared in DMSO (final concentration 0.1-0.2%), added 1 h before stimulation of the cells, and were present during the entire 48 h stimulation period. The effects of test compound on the levels of various biomarkers including cytokines or growth factors, expression of surface molecules, and cell proliferation were subsequently measured. The levels of protein biomarkers were measured by ELISA. Proliferation of PBMC (T cells) was quantified by Alamar blue reduction and proliferation of adherent cell types was quantified by Sulforhodamine B (SRB) assay. Measurement values for each biomarker readout in an exposed sample were divided by the mean value from multiple DMSO control samples to generate a ratio. All ratios were then log10 transformed in the results presentation. Technical details on the Biomap analysis has been published elsewhere [19, 26–27].

### PBMC (T-cell) clustering

Label-free detection of T cell aggregation was performed using the IncuCyte Zoom (Essen Bioscience). Activation of PBMCs with anti-CD3 antibody and IL-2 induces T cell aggregation or “clustering”. Peripheral blood mononuclear cells (PBMC) from healthy donors were isolated by Ficoll Histopaque (Sigma) density gradient centrifugation and stored in aliquots of 30 million cells in −150°C, in FBS supplemented with 10% DMSO. For the experiment PBMC were thawed and diluted in medium RPMI-1640 (Sigma). CD-3 antibody, human IL-2 or medium were added to a 96 well flatbottom plate (50 L per well) to reach a final assay concentration of 0.5 (CD3) and 2.5 (IL-2) M. Drugs were transferred by Echo550 (Labcyte, San Jose, Ca, USA) to a separate empty 96-well flatbottom plate to which PBMCs are added in 200 L medium per well. PBMCs (25 000 cells per well) and drugs were subsequently added to the reagent containing plate to a final volume of 150 L. The plate was then placed in the IncuCyte Zoom and taking phase contrast every 2 hours. Clustering was quantified in the IncuCyte using the on-board software which enables accurate quantification of cell clustering over time (orange mask).

### Measurement of cytokines

The supernatant content of cytokines were measured using the Luminex/MAGPIX system and commercially available kits for various analytes (Biorad, Hercules, CA, USA; R&D, Minneapolis, MN, USA; Millipore, Burlington, MA, USA). The assay is based on binding of the target of interest via antibodies to magnetic beads. The target is detected using biotinylated antibodies with a fluorescent reporter. The assays were performed according to the manufacturer instructions. Briefly, for the cytokine assay, the supernatant samples were incubated first with beads, then with detection antibody and finally with streptavidin-PE. The fluorescence was measured using the MagPix instrument (BioRad) and the concentration levels were calculated by fitting a standard curve.

### Measurement of PBMC induced tumour cell killing

A549NucLightRed (2500 cells per well, 100L) were seeded in a 96 well plate ( Nunc #167008) and placed in an IncucyteZoom (Essen Bioscience) for monitoring phase contrast, red and green fluorescence, taking 4 images of each well at 2-4 hour intervals. After 24 hours or when the cells reached a density of 20%, medium from all the wells were aspirated with EL405 (BioTek instruments, Winooski, BT, USA). Reagents were added in 50 L medium in each well to reach a final assay concentration of Cas 3/7 (Essen Bioscience) 2.5 M, CD-3 (Affymetrix eBioscience, ThermoFisher Scientific) 83.3 ng/mL and human IL-2 (Peprotech) 10 ng/mL. Drugs and vehicle (DMSO) were transferred to an empty 96 well plate (Nunc) by Echo550 (Labcyte) to which PBMCs were added in 200 L medium. PBMCs (25 000 cells per well) and drugs were subsequently added to the reagent and A549 containing plate to a final volume of 150 L. The plates were once again placed in the IncuCyte for monitoring of red (A549 cells) and green (caspase positive cells) object counts.

### Data analysis and statistics

Data was analysed and plotted using GraphPad Prism7 (GraphPad Software Inc., San Diego, CA, USA). Data were processed using the built in Area Under the Curve (AUC) algorithm. Statistical analysis was performed using the paired Students t-test module in GraphPadPrism. The level of statistical significance was set at P < 0.05.

## SUPPLEMENTARY MATERIALS FIGURES AND TABLE



## References

[1] Panni RZ, Linehan DC, DeNardo DG (2013). Targeting tumor-infiltrating macrophages to combat cancer. Immunotherapy.

[2] Fridlender ZG, Albelda SM (2013). Modifying tumor-associated macrophages: an important adjunct to immunotherapy. OncoImmunology.

[3] Stewart DA, Yang Y, Makowski L, Troester MA (2012). Basal-like breast cancer cells induce phenotypic and genomic changes in macrophages. Mol Cancer Res.

[4] Pantziarka P, Bouche G, Meheus L, Sukhatme V, Sukhatme VP (2014). Repurposing Drugs in Oncology (ReDO)-mebendazole as an anti-cancer agent. Ecancermedicalscience.

[5] Doudican N, Rodriguez A, Osman I, Orlow SJ (2008). Mebendazole induces apoptosis via Bcl-2 inactivation in chemoresistant melanoma cells. Mol Cancer Res.

[6] Bai RY, Staedtke V, Aprhys CM, Gallia GL, Riggins GJ (2011). Antiparasitic mebendazole shows survival benefit in 2 preclinical models of glioblastoma multiforme. Neuro-oncol.

[7] Sasaki J, Ramesh R, Chada S, Gomyo Y, Roth JA, Mukhopadhyay T (2002). The anthelmintic drug mebendazole induces mitotic arrest and apoptosis by depolymerizing tubulin in non-small cell lung cancer cells. Mol Cancer Ther.

[8] Martarelli D, Pompei P, Baldi C, Mazzoni G (2008). Mebendazole inhibits growth of human adrenocortical carcinoma cell lines implanted in nude mice. Cancer Chemother Pharmacol.

[9] Mukhopadhyay T, Sasaki J, Ramesh R, Roth JA (2002). Mebendazole elicits a potent antitumor effect on human cancer cell lines both in vitro and in vivo. Clin Cancer Res.

[10] Nygren P, Fryknäs M, Agerup B, Larsson R (2013). Repositioning of the anthelmintic drug mebendazole for the treatment for colon cancer. J Cancer Res Clin Oncol.

[11] Doudican NA, Byron SA, Pollock PM, Orlow SJ (2013). XIAP downregulation accompanies mebendazole growth inhibition in melanoma xenografts. Anticancer Drugs.

[12] Bai RY, Staedtke V, Rudin CM, Bunz F, Riggins GJ (2015). Effective treatment of diverse medulloblastoma models with mebendazole and its impact on tumor angiogenesis. Neuro-oncol.

[13] Bodhinayake I, Symons M, Boockvar JA (2015). Repurposing mebendazole for the treatment of medulloblastoma. Neurosurgery.

[14] Pinto LC, Soares BM, Pinheiro JJ, Riggins GJ, Assumpção PP, Burbano RM, Montenegro RC (2015). The anthelmintic drug mebendazole inhibits growth, migration and invasion in gastric cancer cell model. Toxicol In Vitro.

[15] Dobrosotskaya IY, Hammer GD, Schteingart DE, Maturen KE, Worden FP (2011). Mebendazole monotherapy and long-term disease control in metastatic adrenocortical carcinoma. Endocr Pract.

[16] Nygren P, Larsson R (2014). Drug repositioning from bench to bedside: tumour remission by the antihelmintic drug mebendazole in refractory metastatic colon cancer. Acta Oncol.

[17] Larsen AR, Bai RY, Chung JH, Borodovsky A, Rudin CM, Riggins GJ, Bunz F (2015). Repurposing the antihelmintic mebendazole as a hedgehog inhibitor. Mol Cancer Ther.

[18] Blom K, Senkowski W, Jarvius M, Berglund M, Rubin J, Lenhammar L, Parrow V, Andersson C, Loskog A, Fryknäs M, Nygren P, Larsson R (2017). The anticancer effect of mebendazole may be due to M1 monocyte/macrophage activation via ERK1/2 and TLR8-dependent inflammasome activation. Immunopharmacol Immunotoxicol.

[19] Berg EL, Yang J, Melrose J, Nguyen D, Privat S, Rosler E, Kunkel EJ, Ekins S (2010). Chemical target and pathway toxicity mechanisms defined in primary human cell systems. J Pharmacol Toxicol Methods.

[20] Braithwaite PA, Roberts MS, Allan RJ, Watson TR (1982). Clinical pharmacokinetics of high dose mebendazole in patients treated for cystic hydatid disease. Eur J Clin Pharmacol.

[21] Cui S, Reichner JS, Mateo RB, Albina JE (1994). Activated murine macrophages induce apoptosis in tumor cells through nitric oxide-dependent or -independent mechanisms. Cancer Res.

[22] Biswas SK, Mantovani A (2010). Macrophage plasticity and interaction with lymphocyte subsets: cancer as a paradigm. Nat Immunol.

[23] Tanaka H, Matsushima H, Mizumoto N, Takashima A (2009). Classification of chemotherapeutic agents based on their differential in vitro effects on dendritic cells. Cancer Res.

[24] Tanaka H, Matsushima H, Nishibu A, Clausen BE, Takashima A (2009). Dual therapeutic efficacy of vinblastine as a unique chemotherapeutic agent capable of inducing dendritic cell maturation. Cancer Res.

[25] Mizuno K, Toyoda Y, Fukami T, Nakajima M, Yokoi T (2011). Stimulation of pro-inflammatory responses by mebendazole in human monocytic THP-1 cells through an ERK signaling pathway. Arch Toxicol.

[26] Bergamini G, Bell K, Shimamura S, Werner T, Cansfield A, Müller K, Perrin J, Rau C, Ellard K, Hopf C, Doce C, Leggate D, Mangano R (2012). A selective inhibitor reveals PI3Kγ dependence of T(H)17 cell differentiation. Nat Chem Biol.

[27] Kunkel EJ, Plavec I, Nguyen D, Melrose J, Rosler ES, Kao LT, Wang Y, Hytopoulos E, Bishop AC, Bateman R, Shokat KM, Butcher EC, Berg EL (2004). Rapid structure-activity and selectivity analysis of kinase inhibitors by BioMAP analysis in complex human primary cell-based models. Assay Drug Dev Technol.

