# Supplementary material for: Mebendazole stimulates CD14+ myeloid cells to enhance T-cell activation and tumour cell killing
Source: Oncotarget. 2018 Jul 20;9(56):30805–13. doi: 10.18632/oncotarget.25713 (PMC6089388; doi:10.18632/oncotarget.25713)
Supplement: Supplementary file 1 [file oncotarget-09-30805-s001.pdf]

Mebendazole stimulates CD14+ myeloid cells to enhance T-cell activation and tumour cell killing

SUPPLEMENTARY MATERIALS

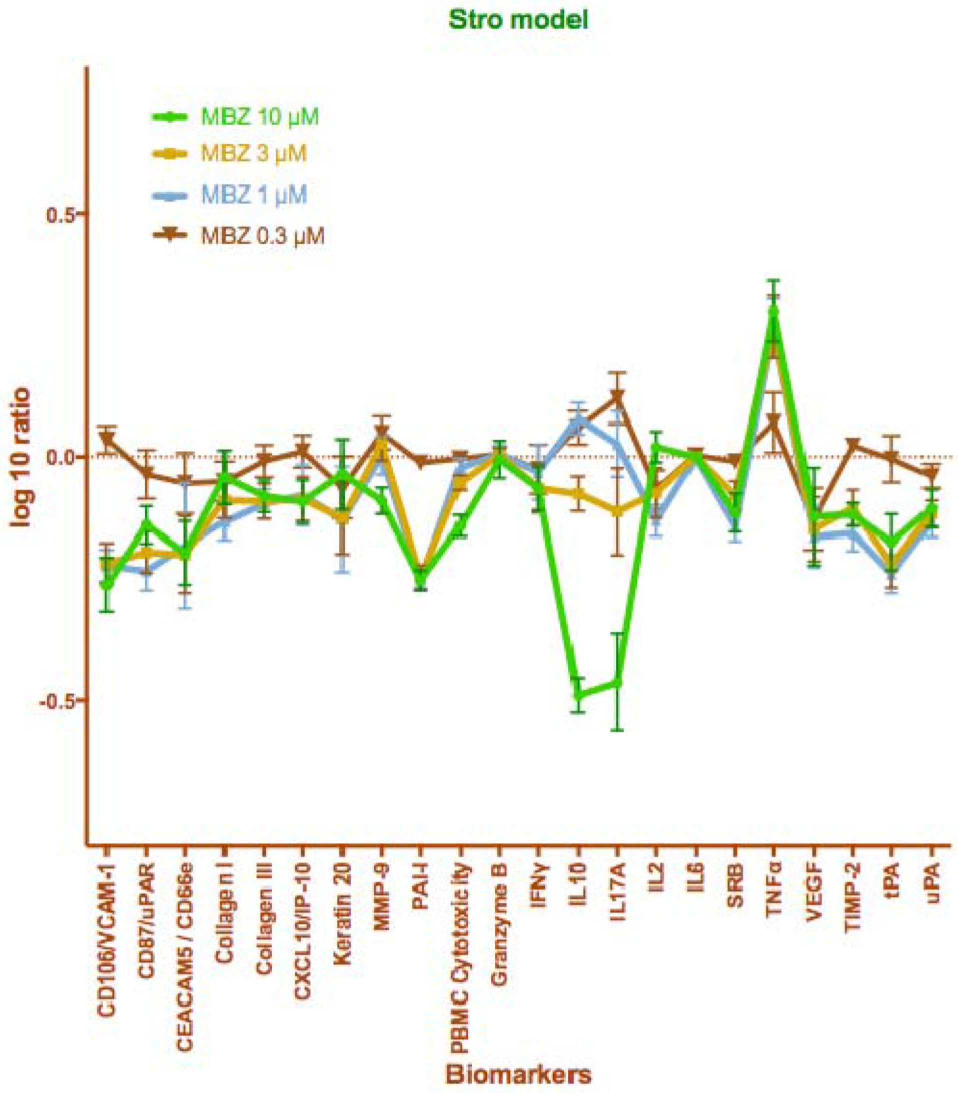

Supplemenatry Figure 1: Biomap profiles of MBZ, tested at multiple concentrations in 1 BioMAP systems, Stro model. The biomarker readouts measured (see Methods) are indicated along the x-axis. The y-axis shows the log10 expression ratios of the readout level measurements relative to solvent (DMSO buffer) controls. Each datapoint represents a single well.

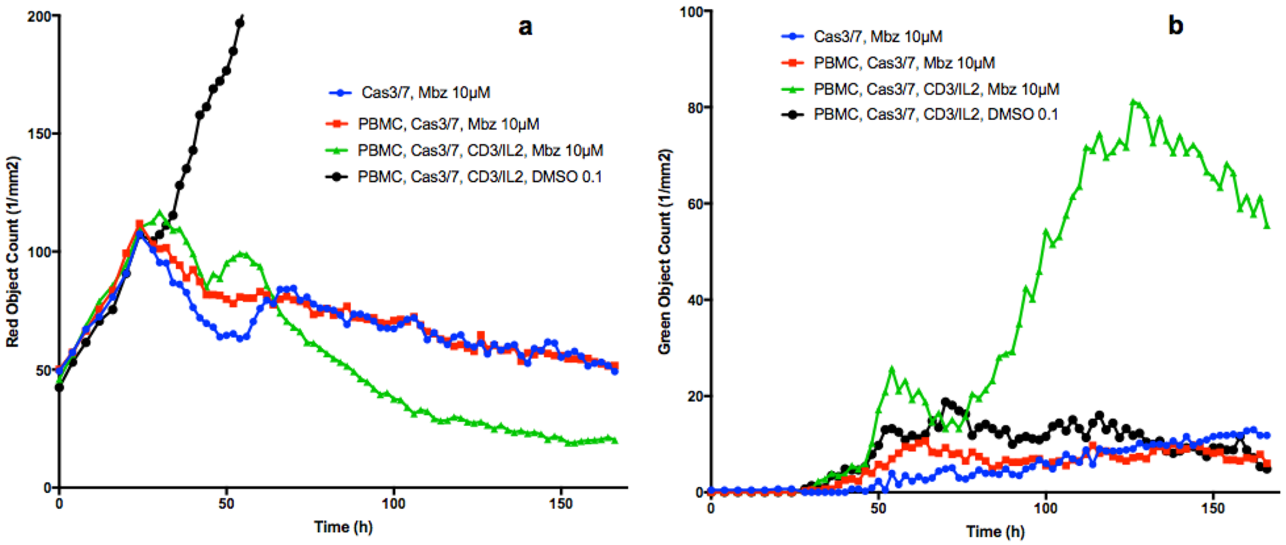

Supplemenatry Figure 2: Co-culture of PBMC and red fluorescence-labeled A549 lung cancer cells in medium containing caspase 3/7 probe (Cas3/7). The kinetics of changes in A549 cell survival (red object count) and apoptosis (green object count, cas3/7 positive cells) in response to anti-CD3/IL2 and MBZ (10 µM) is presented in panel **(a)** and **(b)**, respectively. Statiscal analysis is presented in Supplementary Table 1.

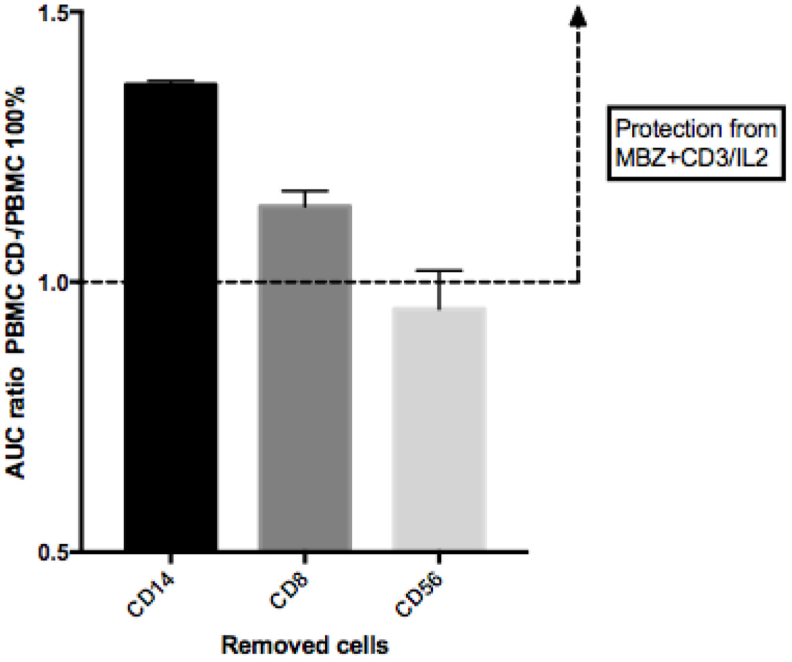

Supplemenatry Figure 3: Effect of removal of CD14, CD8 and CD56 cells on MBZ induced A549Red cell kill in CD3/ IL2 treated cells. Results are presened as mean AUC ratios for depleted and intact PBMC cultures (n=2) treated with MBZ and antiCD3/ IL2 as described in material and methods.

Supplementary Table 1: Statistical analysis of the differences in AUC (MBZ 10 µM)

Object (AUC)	Comparison	Mean Difference	P-value	N	PVS[1]
**Red**	DMSO+PBMC CD3/IL2 vs. MBZ+PBMC CD3/IL2	22912	0.0005	6	***
**Red**	MBZ+PBMC vs. MBZ+PBMC CD3/IL2	2645	0.0007	6	***
**Red**	MBZ vs. MBZ+PBMC CD3/IL2	2568	0.0012	6	**
**Green**	DMSO+PBMC CD3/IL2 vs. MBZ+PBMC CD3/IL2	2696	0.0246	5	*
**Green**	MBZ+PBMC vs. MBZ+PBMC CD3/IL2	4626	0.0391	5	*
**Green**	MBZ vs. MBZ+PBMC CD3/IL2	4738	0.0183	5	*

Data was analysed by two-tailed paired t test using Graphpad Prism. [1]P value summary.
